# A real-world pharmacovigilance study of Sorafenib based on the FDA Adverse Event Reporting System

**DOI:** 10.3389/fphar.2024.1442765

**Published:** 2024-12-17

**Authors:** Dongdong Zhang, Ying Cai, Yixin Sun, Peiji Zeng, Wei Wang, Wenhui Wang, Xiaohua Jiang, Yifan Lian

**Affiliations:** ^1^ Department of Gastroenterology, Zhongshan Hospital of Xiamen University, School of Medicine, Xiamen University, Xiamen, Fujian, China; ^2^ Department of Digestive Disease, School of Medicine, Institute for Microbial Ecology, Xiamen University, Xiamen, Fujian, China; ^3^ School of Medicine, Xiamen University, Xiamen, Fujian, China; ^4^ Department of Orthopedics, Xiang’an Hospital of Xiamen University, School of Medicine, Xiamen University, Xiamen, Fujian, China

**Keywords:** Sorafenib, FAERS, ROR, BCPNN, EBGM

## Abstract

**Aims:**

The primary objective of this study was to closely monitor and identify adverse events (AEs) associated with Sorafenib, a pharmacological therapeutic agent used to treat hepatocellular carcinoma, renal cell carcinoma, and thyroid cancer. The ultimate goal was to optimize patient safety and provide evidence-based guidance for the appropriate use of this drug.

**Methods:**

Reports from the FDA Adverse Event Reporting System (FAERS) database were comprehensively collected and analyzed, covering the first quarter of 2004 to the first quarter of 2024. Disproportionality analysis was performed using robust algorithms for effective data mining to quantify the signals associated with Sorafenib-related AEs.

**Results:**

In total, we identifued 18,624 patients (82,857 AEs in the Sorafenib population) from the collected reports and examined, the occurrence of Sorafenib-induced AEs in 26 organ systems. The study results revealed the presence of the expected AEs, including Diarrhoea, Palmar-plantar erythrodysaesthesia syndrome, Hepatocellular carcinoma, Fatigue, and Rash, which was consistent with the information provided in the drug insert. In addition, unexpected significant AEs, such as Gait inability, Palmoplantar keratoderma and Hyperkeratosis were observed at the preferred term (PT) level. These findings suggest the potential occurrence of adverse reactions not currently documented in drug descriptions.

**Conclusion:**

This study successfully detected new and unforeseen signals associated with Sorafenib-related AEs related to Sorafenib administration, providing important insights into the complex correlations between AEs and Sorafenib use. The results of this study emphasize the critical importance of continuous and vigilant surveillance for the timely identification and effective management of AEs to improve the overall patient safety and wellbeing in the context of Sorafenib therapy.

## Introduction

Sorafenib is a novel multi-targeted antitumor drug that acts on both tumor cells and tumor vasculature (Siegel et al., 2010). It has been approved by the U.S. Food and Drug Administration (FDA) for the treatment of hepatocellular carcinoma (HCC), advanced renal cell carcinoma (RCC), and differentiated thyroid carcinoma in patients refractory to radioactive iodine (Chang et al., 2007; Hsieh et al., 2017; Boucai et al., 2024; Cappuyns et al., 2024). These illnesses show a large worldwide burden. Thyroid cancer affects about 60,000 people in the US each year, and HCC is the sixth most common neoplasm and the third leading cause of cancer death. In 2020, the global age-standardized incidence rate (ASR) of RCC was 4.6 per 100,000 people, making it the 16th most common cancer (Haugen et al., 2016; Hsieh et al., 2017; Forner et al., 2018; Villanueva, 2019).

Sorafenib has dual antitumor effects, as it can directly inhibit tumor cell proliferation by blocking the cell signaling pathway mediated by RAF/MEK/ERK (Wilhelm et al., 2004; Liu et al., 2006) and indirectly inhibit tumor cell growth by blocking tumor neovascularization through the inhibition of vascular endothelial growth factor receptor (VEGFR) and platelet-derived growth factor (PDGF) receptors (Wilhelm et al., 2008; Capdevila et al., 2024). The relevance of tracking adverse events (AEs) in real-world data is suggested by the widespread use of Sorafenib in the clinical context and its tolerable levels of toxicity. The disparity between the current high use of Sorafenib and our knowledge of its safety is highlighted by case reports showing that a greater number of AEs are linked to the use of Sorafenib as monotherapy or in combination with other medications (Celsa et al., 2024; Pitoia et al., 2024). Therefore, it is critical to research and evaluate the safety of Sorafenib in actual clinical practice.

FDA Adverse Event Reporting System (FAERS) is a quintessential public self-reporting system that collects all spontaneous safety reports and post-marketing clinical studies related to the use of FDA-approved drugs and therapeutic biologics within and outside the United States, and has been used extensively for screening drug safety information (Su et al., 2023), and investigate the safety and efficacy of Sorafenib-related therapy. Our study makes use of a thorough disproportionate analysis of Sorafenib-related AEs in real-world data extracted from the FAERS database. By closely examining the signal strength in real-world data, this analysis can help to effectively detect and manage AEs. Our study aimed to contribute to the evidence of Sorafenib AEs and clarify their safety by assessing these data. This study highlights the value of continuing post-marketing drug safety monitoring while improving our knowledge of Sorafenib safety. We anticipate that this pharmacovigilance investigation will provide valuable insights into steering therapeutic choices and ultimately protecting patient wellbeing.

## Methodology

### Study design and data sources

**FIGURE 1:**
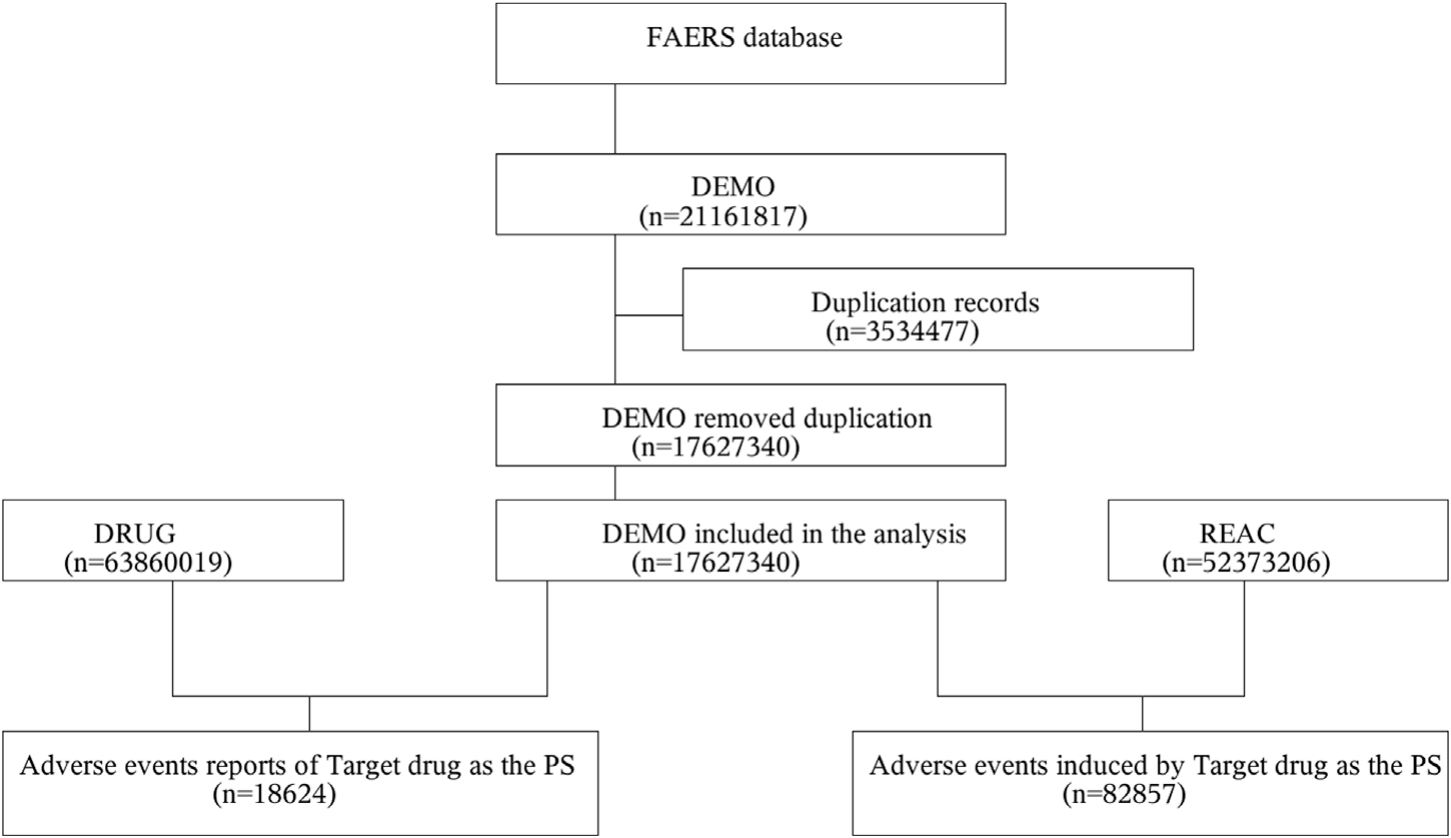
Multistep process of data extraction, processing, and analysis from the Food and Drug Administration adverse event reporting system database.

This study was designed as an observational, retrospective disproportionality analysis, a validated concept in pharmacovigilance, to assess whether an association exists between Sorafenib and AEs. The FAERS data were downloaded from the FAERS Quarterly Data Extract Files, available at https://fis.fda.gov/extensions/FPD-QDE-FAERS/FPD-QDE-FAERS html. The raw data used for data mining were 81 quarters from Q1-2004 to Q1-2024, with a total of 17, 627, 340 background patients (5,237,3206 AEs) included in the analysis, of which the number of patients in the population of the target drug Sorafenib was 18,624 (82,857 AEs). Because the FAERS database collects data by spontaneous submission, few duplicate reports or withdrawn/deleted reports were present in the database, and data cleaning in this study was conducted in strict accordance with the guidance document on the official website of the FDA. The rules of data cleaning were, first to deduplicate reports according to the FDA-recommended method of removing duplicate reports, selecting the PRIMARYID, CASEID, and FDA_DT fields of the DEMO table, sorting them according to CASEID, FDA_DT, and PRIMARYID, and keeping the one with the largest value of FDA_DT for the reports with the same CASEID and FDA_DT for reports with the same CASEID and FDA_DT. And FDA_DT values were the same as those with the highest PRIMARYID values. Second, a list of deleted reports exists in each quarterly packet from Q1-2019 to, and after which reports were excluded based on the CASEID in the list of deleted reports. To identify adverse drug reactions (ADRs), statistical methods were employed. This study collected incidences of Sorafenib-related, and the AEs were classified into preferred terms (PTs) and system organ classes (SOCs) reflecting different levels of the Medical Dictionary for Regulatory Activities (MedDRA). A data-screening flowchart is shown in Figure 1.

### Statistical analysis

Pharmacovigilance now performs a disproportionality analysis to identify possible drug-AE correlations. Four methods were used for the disproportionality analysis: reporting odds ratio (ROR), proportional reporting ratio (PRR), Bayesian confidence propagation neural network (BCPNN) (Lindquist et al., 2000; Sun et al., 2019), and multi-item gamma Poisson shrinkage (MGPS). Four algorithms were applied to quantify the AE signal associated with Sorafenib, and their equations and criteria are listed in Supplementary Table S1. Extraction decision rules for these algorithms were applied to detect signals and scores were calculated to assess drug and AE. If at least one of the four indices met the criteria, a signal would be detected at the system organ class (SOC) level. If all four indices met the criteria, a signal would be detected at the PT level (Sakaeda et al., 2013; Hu et al., 2020). In general, the higher the score for each of the four parameters, the greater the disproportionality (Guo et al., 2022). The statistical analysis was conducted using the SAS software recommended by the FDA official website. After downloading the original ASCII data package from the FDA official website and importing it into SAS9.4 software, the original data were deduplicated according to the FDA’s recommended deduplication rules, and then the data were statistically analyzed.

## Results

### General characteristics

**FIGURE 2:**
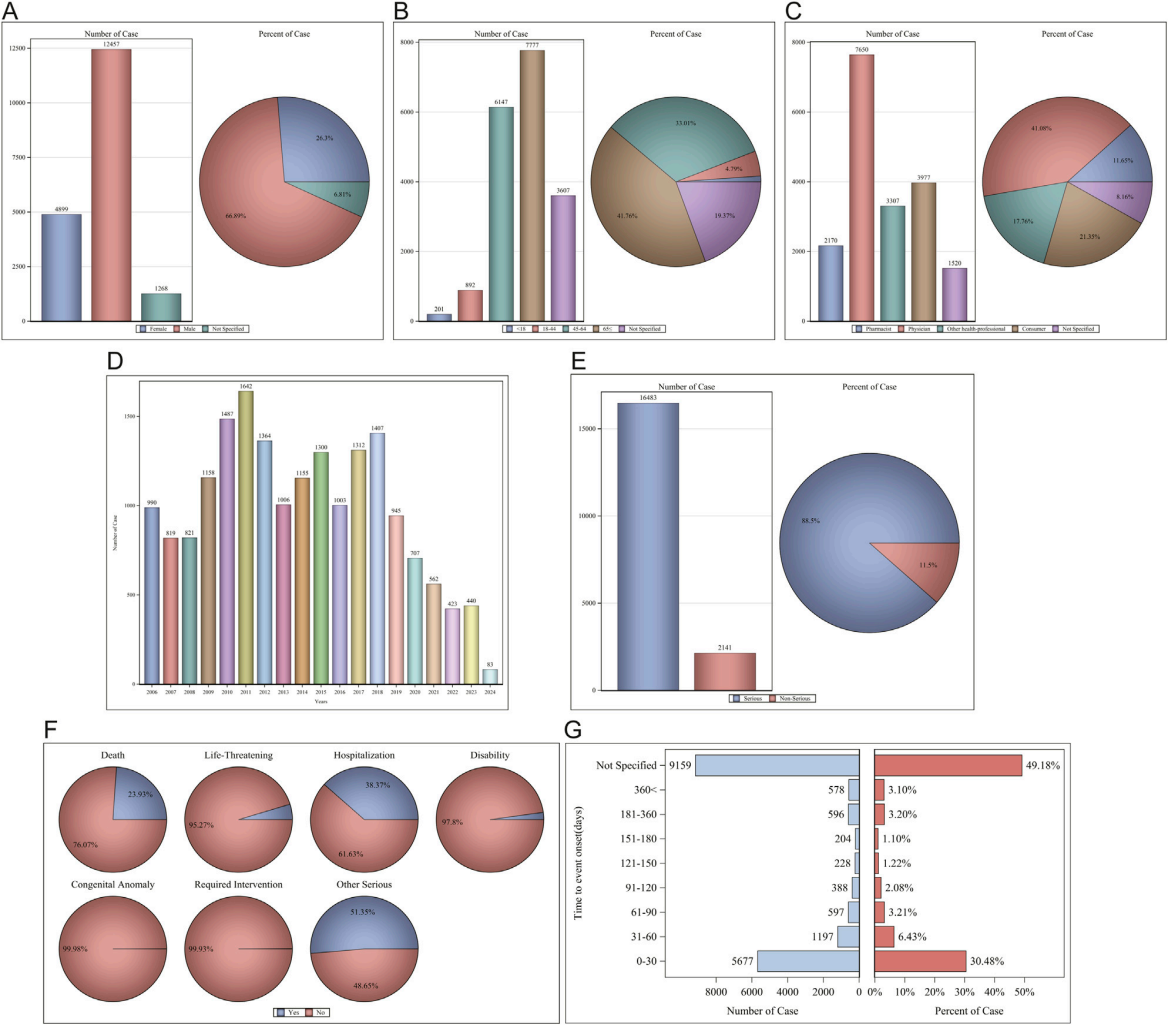
Clinical characteristics of Sorafenib associated reports from the FAERS database. **(A)** Gender; **(B)** Age; **(C)** Reporters; **(D)** Years; **(E)** Serious reports; **(F)** Outcomes; **(G)** AE occurrence time-medication date.

In the FAERS database, a total of 18,624 patients were identified as experiencing 82,857 AEs attributable to Sorafenib, with a mean of 4.4 AEs per person. Among them, 4,899 (26.30%) were female, 12,457 (66.89%) were male, and 1,268 (6.81%) were unspecified for gender (Figure 2A). Age data were available for 15,017 patients (mean age = 63.53 ± 13.51 years); less than 1.1% (1.08%) of patients were under the age of 18 years (n = 201), 4.79% were aged 18–44 years (n = 892), 33.01% were aged 45–64 years (n = 6,147), with the largest proportion of patients being aged ≤65 years (n = 7,777, 41.76%). Age information was not reported for 3,607 patients (Figure 2B). The reporters were primarily physicians and consumers, accounting for 62.43% of the total number of reported figures (Figure 2C). The number of reports was higher between 2009-2018, after which a significant decline was observed (Figure 2D). Serious reports accounted for 88.50% of all reports (Figure 2E). Outcomes were mainly hospitalization-Initial or-prolonged (n = 7,146, 38.37%), death (n = 4,456, 23.93%), and other (n = 9,564, 51.35%) (Figure 2F). The AE occurrence time-medication date, excluding not specified, was mainly concentrated in 0–30 d (30.48%) (Figure 2G). Regarding concomitant medications, there were 45,181 reports of Fulvestrant being used concomitantly with other drugs, with the most frequently concomitant drug being FUROSEMIDE (n = 1,066), followed by AMLODIPINE (n = 1,066). The rankings of the top ten concomitant medications are presented in Table 1; for additional details, please refer to Supplementary Table S2.

**TABLE 1:** The ranking of the top 10 concomitant medications.

Base name_EN	Reports, N
Various	1461
Furosemide	1066
Amlodipine	975
Acetylsalicylic acid	748
Omeprazole	736
Spironolactone	698
Levothyroxine	657
Oxycodone	604
Ursodeoxycholic acid	580
Paracetamol	540

**FIGURE 3:**
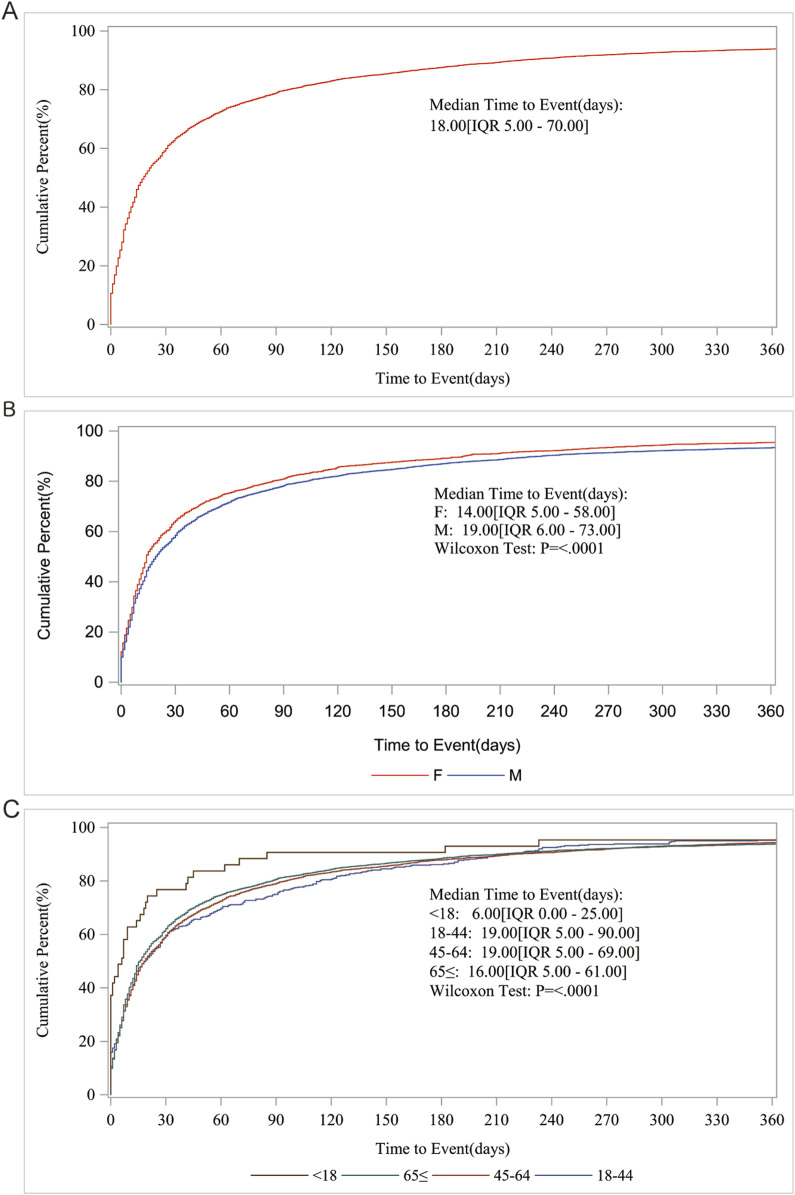
AE occurrence time-medication date subgroup analysis. **(A)** Median Time to Event (days); **(B)** Gender; **(C)** Age.

For the AE occurrence time-medication date, we performed further subgroup analyses and found that the median AE occurrence time-medication date was 18 d, and both sex and age subgroups showed significant differences (*p* < 0001) on the Wilcoxon Test (Figure 3).

Immediately after we analyzed the SOC of Sorafenib AEs, gastrointestinal disorders, general disorders and administration site conditions, skin and subcutaneous tissue disorders were the top three involved, reaching 12,822 (15.47%), 11,248 (13.58%), and 10,673 (12.88%) cases, respectively. The number of cases and percentage of cases exceeded 10,000% and 12%, respectively (Supplementary Figure S1).

### AEs profiling of Sorafenib in disproportionality analysis

**FIGURE 4:**
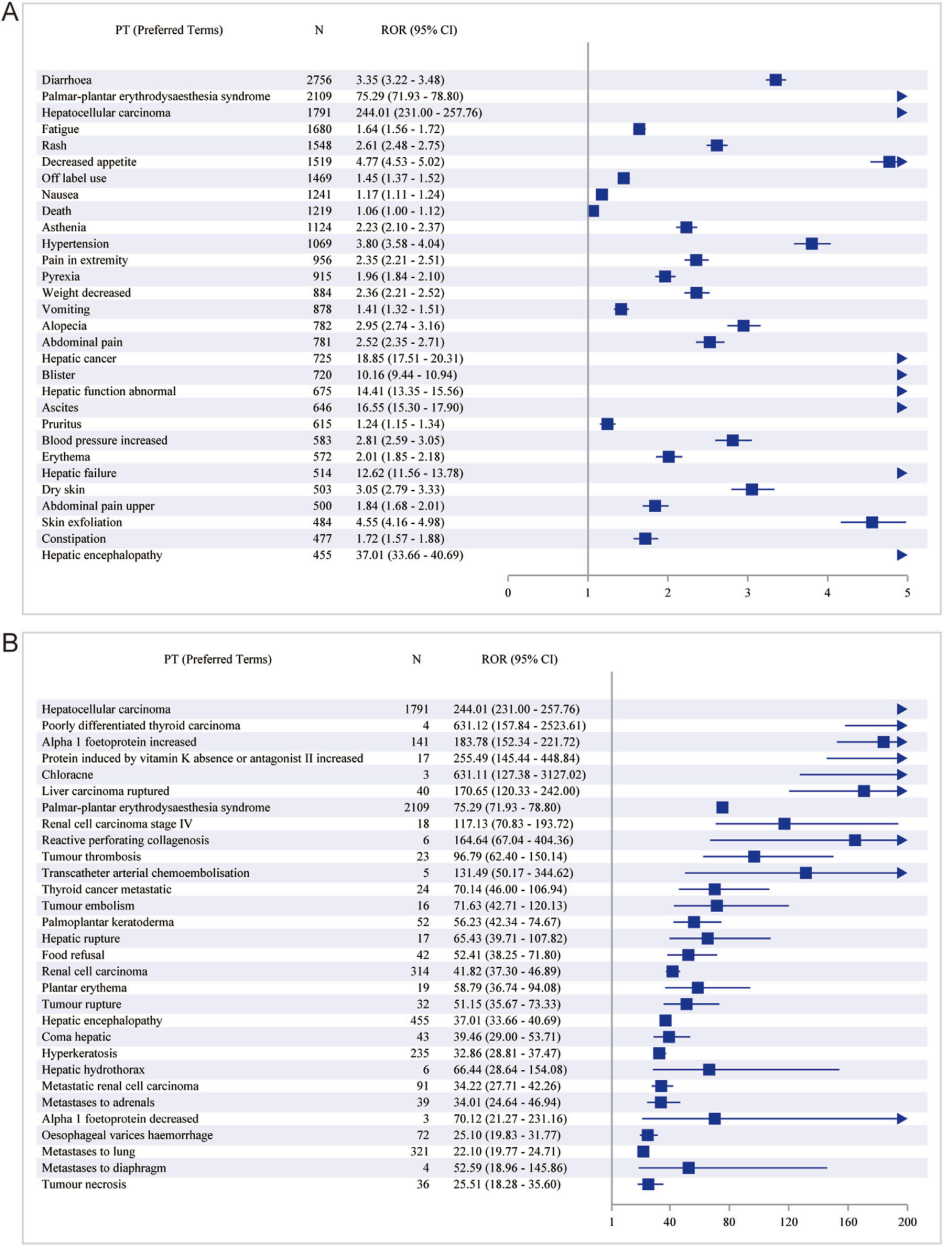
The top 30 PT results for Sorafenib positivity in ROR. **(A)** The highest signal frequency; **(B)** The highest signal intensity.

We preferred the ROR method for signal detection to rank the frequency and intensity of Sorafenib-positive signals. The top 30 PT results are shown in Figure 4. The top five AEs with high frequency of positive signals were diarrhoea [case numbers: 2756, ROR (95% CI) = 3.35 (3.22-3.48)], palmar-plantar erythrodysaesthesia syndrome [case numbers: 2109, ROR (95% CI) = 75.29 (71.93-78.80)], HCC [case numbers. 1791, ROR (95% CI) = 244.01 (231.00-257.76)], fatigue [case numbers: 1,680, ROR (95% CI) = 1.64 (1.56-1.72)] and rash (case numbers: 1,548, ROR (95% CI) = 2.61 (2.48-2.75)] (Figure 4A). The top five AEs with high intensity of positive signals were HCC [case numbers: 1791, ROR (95% CI) = 244.01 (231.00-257.76)], poorly differentiated thyroid carcinoma (case numbers: 4, ROR (95% CI) = 631.12 (157.84-2523.61)], Alpha 1 foetoprotein increased [case numbers: 141, ROR (95% CI) = 183.78 (152.34-221.72)], protein induced by vitamin K absence or antagonist II increased [case numbers: 17, ROR (95% CI) = 255.49 (145.44-448.84)] and chloracne [case numbers: 3, ROR (95% CI) = 631.11 (127.38-3,127.02)] (Figure 4B).

The results of the BCPNN method alone revealed that the top five with high frequency of positive signals were diarrhoea [case numbers: 2756, ROR (95% CI) = 1.70 (1.65-1.76)], palmar-plantar erythrodysaesthesia syndrome [case numbers: 2109, ROR (95% CI) = 6.04 (5.93-6.06)], HCC [case numbers: 1791, ROR (95% CI) = 7.44 (7.23-7.38)], fatigue [case numbers: 1,680, ROR (95% CI) = 0.70 (0.63-0.77)] and rash (case numbers: 1,548, ROR (95% CI) = 1.36 (1.29-1.44) (Table 2). The top five with high intensity of positive signals were HCC [case numbers: 1791, ROR (95% CI) = 7.44 (7.23-7.38)], palmar-plantar erythrodysaesthesia syndrome [case numbers: 2109, ROR (95% CI) = 6.04 (5.93-6.06)], Alpha 1 foetoprotein increased [case numbers: 141, ROR (95% CI) = 7.15 (5.89-6.42)], RCC [case numbers: 314, ROR (95% CI) = 5.29 (4.96-5.29)] and hepatic encephalopathy [case numbers: 455, ROR (95% CI) = 5.12 (4.88-5.16)] (Supplementary Table S3).

**TABLE 2:** The top 30 PTs with the highest signal frequency for Sorafenib positivity in BCPNN.

Preferred terms	Case	IC (95% CI)
Diarrhoea	2756	1.70 (1.65–1.76)
Palmar-plantar erythrodysaesthesia syndrome	2109	6.04 (5.93–6.06)
Hepatocellular carcinoma	1791	7.44 (7.23–7.38)
Fatigue	1680	0.70 (0.63–0.77)
Rash	1548	1.36 (1.29–1.44)
Decreased appetite	1519	2.22 (2.15–2.30)
Off label use	1469	0.52 (0.45–0.60)
Nausea	1241	0.23 (0.14–0.31)
Death	1219	0.08 (0.00–0.17)
Asthenia	1124	1.14 (1.06–1.23)
Hypertension	1069	1.91 (1.81–1.99)
Pain in extremity	956	1.22 (1.13–1.31)
Pyrexia	915	0.96 (0.87–1.06)
Weight decreased	884	1.23 (1.13–1.32)
Vomiting	878	0.49 (0.39–0.59)
Alopecia	782	1.55 (1.44–1.65)
Abdominal pain	781	1.32 (1.22–1.42)
Hepatic cancer	725	4.18 (4.04–4.26)
Blister	720	3.31 (3.19–3.40)
Hepatic function abnormal	675	3.81 (3.67–3.89)
Ascites	646	4.00 (3.85–4.09)
Pruritus	615	0.31 (0.19–0.43)
Blood pressure increased	583	1.48 (1.36–1.60)
Erythema	572	1.00 (0.88–1.12)
Hepatic failure	514	3.62 (3.46–3.72)
Dry skin	503	1.60 (1.46–1.72)
Abdominal pain upper	500	0.87 (0.74–1.00)
Skin exfoliation	484	2.17 (2.03–2.29)
Constipation	477	0.77 (0.64–0.90)
Hepatic encephalopathy	455	5.12 (4.88–5.16)

Of course, for more accurate and objective results, we combined ROR and PRR to detect the signals, and compared the results with the ROR test results alone, fatigue [case numbers: 1,680, ROR (95% CI) = 1.64 (1.56-1.72)] in the first five PTs of the frequency of positive signals was replaced by decreased appetite [case numbers: 1,519, ROR (95% CI) = 4.77 (4.53-5.02), PRR (Chi-Square) = 4.70 (4,411.64)] (Table 3). A small difference was noted in the intensities of the positive signals (Supplementary Table S4). In contrast, the results of the combined ROR and BCPNN methods were similar to tha of the ROR assay alone (Supplementary Tables S5, S6).

**TABLE 3:** The top 30 PTs with the highest signal frequency for Sorafenib positivity in ROR and PRR.

Preferred terms	Case	ROR (95% CI)	PRR (Chi-Square)
Diarrhoea	2756	3.35 (3.22–3.48)	3.27 (4367.98)
Palmar-plantar erythrodysaesthesia syndrome	2109	75.29 (71.93–78.80)	73.40 (134966)
Hepatocellular carcinoma	1791	244.01 (231.00–257.76)	238.76 (307689)
Rash	1548	2.61 (2.48–2.75)	2.58 (1504.22)
Decreased appetite	1519	4.77 (4.53–5.02)	4.70 (4411.64)
Asthenia	1124	2.23 (2.10–2.37)	2.21 (750.06)
Hypertension	1069	3.80 (3.58–4.04)	3.76 (2164.83)
Pain in extremity	956	2.35 (2.21–2.51)	2.34 (732.79)
Weight decreased	884	2.36 (2.21–2.52)	2.34 (680.98)
Alopecia	782	2.95 (2.74–3.16)	2.93 (990.84)
Abdominal pain	781	2.52 (2.35–2.71)	2.51 (708.33)
Hepatic cancer	725	18.85 (17.51–20.31)	18.70 (11800.5)
Blister	720	10.16 (9.44–10.94)	10.08 (5802.44)
Hepatic function abnormal	675	14.41 (13.35–15.56)	14.30 (8171.70)
Ascites	646	16.55 (15.30–17.90)	16.43 (9126.57)
Blood pressure increased	583	2.81 (2.59–3.05)	2.80 (672.41)
Erythema	572	2.01 (1.85–2.18)	2.00 (286.73)
Hepatic failure	514	12.62 (11.56–13.78)	12.55 (5359.84)
Dry skin	503	3.05 (2.79–3.33)	3.04 (686.28)
Skin exfoliation	484	4.55 (4.16–4.98)	4.53 (1325.18)
Hepatic encephalopathy	455	37.01 (33.66–40.69)	36.81 (14979.3)
General physical health deterioration	425	2.99 (2.72–3.29)	2.98 (558.75)
Platelet count decreased	415	2.89 (2.63–3.19)	2.88 (509.53)
Dysphonia	412	5.26 (4.77–5.80)	5.24 (1403.01)
Dehydration	409	2.23 (2.03–2.46)	2.23 (276.25)
Stomatitis	400	5.10 (4.62–5.62)	5.08 (1300.02)
Hospitalisation	385	2.04 (1.84–2.25)	2.03 (201.61)
Oedema peripheral	352	2.03 (1.83–2.26)	2.03 (183.61)
Metastases to lung	321	22.10 (19.77–24.71)	22.02 (6224.85)
Renal cell carcinoma	314	41.82 (37.30–46.89)	41.67 (11691.9)

Immediately after that, we used the joint detection of the four most stringent algorithms, sorted by frequency (Table 4), with the top five diarrhoea [case numbers: 2756, ROR (95% CI) = 3.35 (3.22-3.48), PRR (Chi-Square) = 3.27 (4,367.98), IC (IC-2SD) = 1.70 (1.65), EBGM (EBGM05) = 3.26 (3.14)], palmar-plantar erythrodysaesthesia syndrome [case numbers: 2109, ROR (95% CI) = 75.29 (71.93-78.80), PRR (Chi-Square) = 73.40 (134,966), IC (IC-2SD) = 6.04 (5.93), EBGM (EBGM05) = 65.85 (62.92)], HCC [case numbers: 1791, ROR (95% CI) = 244.01 (231.00-257.76), PRR (Chi-Square) = 238.76 (307,689), IC (IC-2SD) = 7.44 (7.23), EBGM (EBGM05) = 173.50 (164.25)], rash [case numbers: 1,548, ROR (95% CI) = 2.61 (2.48-2.75), PRR (Chi-Square) = 2.58 (1,504.22), IC (IC-2SD) = 1.36 (1.29), EBGM (EBGM05) = 2.57 (2.45)] and decreased appetite [case numbers: 1,519, ROR (95% CI) = 4.77 (4.53-5.02), PRR (Chi-Square) = 4.70 (4,411.64), IC (IC-2SD) = 2.22 (2.15), EBGM (EBGM05) = 4.67 (4.44)]. In order of positive signal intensity (in descending order of the lower limit of the ROR 95% confidence interval) (Table 5), the top five conditions were HCC [case numbers: 1791, ROR (95% CI) = 244.01 (231.00-257.76), PRR (Chi-Square) = 238.76 (307,689), IC (IC-2SD) = 7.44 (7.23), EBGM (EBGM05) = 173.50 (164.25)], poorly differentiated thyroid carcinoma [case numbers: 4, ROR (95% CI) = 631.12 (157.84-2523.61), PRR (Chi-Square) = 631.09 (1,258.19), IC (IC-2SD) = 8.30 (0.69), EBGM (EBGM05) = 316.05 (79.04)], Alpha 1 foetoprotein increased [case numbers: 141, ROR (95% CI) = 183.78 (152.34-221.72), PRR (Chi-Square) = 183.47 (19,825.1), IC(IC-2SD) = 7.15 (5.89), EBGM (EBGM05) = 142.37 (118.01)], protein induced by vitamin K absence or antagonist II increased [case numbers: 17, ROR (95% CI) = 255.49 (145.44-448.84), PRR (Chi-Square) = 255.44 (3,067.13), IC (IC-2SD) = 7.51 (3.26), EBGM (EBGM05) = 182.13 (103.67)] and chloracne [case numbers: 3, ROR (95% CI) = 631.11 (127.38-3,127.02), PRR (Chi-Square) = 631.09 (943.64), IC (IC-2SD) = 8.30 (0.18), EBGM (EBGM05) = 316.05 (63.79)].

**TABLE 4:** The top 30 PTs with the highest signal frequency for Sorafenib positivity in ROR, PRR, BCPNN, and MGPS.

Preferred terms	Case	ROR (95% CI)	PRR (Chi-Square)	IC (IC-2SD)	EBGM (EBGM05)
Diarrhoea	2756	3.35 (3.22–3.48)	3.27 (4367.98)	1.70 (1.65)	3.26 (3.14)
Palmar-plantar erythrodysaesthesia syndrome	2109	75.29 (71.93–78.80)	73.40 (134966)	6.04 (5.93)	65.85 (62.92)
Hepatocellular carcinoma	1791	244.01 (231.00–257.76)	238.76 (307689)	7.44 (7.23)	173.50 (164.25)
Rash	1548	2.61 (2.48–2.75)	2.58 (1504.22)	1.36 (1.29)	2.57 (2.45)
Decreased appetite	1519	4.77 (4.53–5.02)	4.70 (4411.64)	2.22 (2.15)	4.67 (4.44)
Asthenia	1124	2.23 (2.10–2.37)	2.21 (750.06)	1.14 (1.06)	2.21 (2.08)
Hypertension	1069	3.80 (3.58–4.04)	3.76 (2164.83)	1.91 (1.81)	3.75 (3.53)
Pain in extremity	956	2.35 (2.21–2.51)	2.34 (732.79)	1.22 (1.13)	2.33 (2.19)
Weight decreased	884	2.36 (2.21–2.52)	2.34 (680.98)	1.23 (1.13)	2.34 (2.19)
Alopecia	782	2.95 (2.74–3.16)	2.93 (990.84)	1.55 (1.44)	2.92 (2.72)
Abdominal pain	781	2.52 (2.35–2.71)	2.51 (708.33)	1.32 (1.22)	2.50 (2.33)
Hepatic cancer	725	18.85 (17.51–20.31)	18.70 (11800.5)	4.18 (4.04)	18.19 (16.89)
Blister	720	10.16 (9.44–10.94)	10.08 (5802.44)	3.31 (3.19)	9.94 (9.23)
Hepatic function abnormal	675	14.41 (13.35–15.56)	14.30 (8171.70)	3.81 (3.67)	14.01 (12.98)
Ascites	646	16.55 (15.30–17.90)	16.43 (9126.57)	4.00 (3.85)	16.04 (14.83)
Blood pressure increased	583	2.81 (2.59–3.05)	2.80 (672.41)	1.48 (1.36)	2.79 (2.57)
Hepatic failure	514	12.62 (11.56–13.78)	12.55 (5359.84)	3.62 (3.46)	12.32 (11.29)
Dry skin	503	3.05 (2.79–3.33)	3.04 (686.28)	1.60 (1.46)	3.03 (2.77)
Skin exfoliation	484	4.55 (4.16–4.98)	4.53 (1325.18)	2.17 (2.03)	4.51 (4.12)
Hepatic encephalopathy	455	37.01 (33.66–40.69)	36.81 (14979.3)	5.12 (4.88)	34.84 (31.69)
General physical health deterioration	425	2.99 (2.72–3.29)	2.98 (558.75)	1.57 (1.43)	2.97 (2.70)
Platelet count decreased	415	2.89 (2.63–3.19)	2.88 (509.53)	1.52 (1.38)	2.88 (2.61)
Dysphonia	412	5.26 (4.77–5.80)	5.24 (1403.01)	2.38 (2.22)	5.20 (4.72)
Dehydration	409	2.23 (2.03–2.46)	2.23 (276.25)	1.15 (1.01)	2.22 (2.02)
Stomatitis	400	5.10 (4.62–5.62)	5.08 (1300.02)	2.33 (2.17)	5.04 (4.57)
Metastases to lung	321	22.10 (19.77–24.71)	22.02 (6224.85)	4.41 (4.16)	21.31 (19.06)
Renal cell carcinoma	314	41.82 (37.30–46.89)	41.67 (11691.9)	5.29 (4.96)	39.15 (34.92)
Blood bilirubin increased	296	7.93 (7.07–8.90)	7.91 (1764.33)	2.97 (2.77)	7.82 (6.97)
Gait inability	266	3.44 (3.05–3.88)	3.43 (457.08)	1.77 (1.58)	3.42 (3.03)
Jaundice	266	7.04 (6.23–7.94)	7.02 (1358.07)	2.80 (2.59)	6.95 (6.16)

**TABLE 5:** The top 30 PTs with the highest signal intensity for Sorafenib positivity in ROR, PRR, BCPNN, and MGPS.

Preferred terms	Case	ROR (95% CI)	PRR (Chi-Square)	IC (IC-2SD)	EBGM (EBGM05)
Hepatocellular carcinoma	1791	244.01 (231.00–257.76)	238.76 (307689)	7.44 (7.23)	173.50 (164.25)
Poorly differentiated thyroid carcinoma	4	631.12 (157.84–2523.61)	631.09 (1258.19)	8.30 (0.69)	316.05 (79.04)
Alpha 1 foetoprotein increased	141	183.78 (152.34–221.72)	183.47 (19825.1)	7.15 (5.89)	142.37 (118.01)
Protein induced by vitamin K absence or antagonist II increased	17	255.49 (145.44–448.84)	255.44 (3067.13)	7.51 (3.26)	182.13 (103.67)
Chloracne	3	631.11 (127.38–3127.02)	631.09 (943.64)	8.30 (0.18)	316.05 (63.79)
Liver carcinoma ruptured	40	170.65 (120.33–242.00)	170.57 (5308.22)	7.07 (4.48)	134.49 (94.83)
Palmar-plantar erythrodysaesthesia syndrome	2109	75.29 (71.93–78.80)	73.40 (134966)	6.04 (5.93)	65.85 (62.92)
Renal cell carcinoma stage IV	18	117.13 (70.83–193.72)	117.11 (1747.80)	6.63 (3.29)	98.94 (59.82)
Reactive perforating collagenosis	6	164.64 (67.04–404.36)	164.63 (773.94)	7.03 (1.53)	130.78 (53.25)
Tumour thrombosis	23	96.79 (62.40–150.14)	96.77 (1890.08)	6.39 (3.61)	84.04 (54.18)
Transcatheter arterial chemoembolisation	5	131.49 (50.17–344.62)	131.48 (535.80)	6.77 (1.23)	108.98 (41.58)
Thyroid cancer metastatic	24	70.14 (46.00–106.94)	70.12 (1471.73)	5.98 (3.57)	63.21 (41.46)
Tumour embolism	16	71.63 (42.71–120.13)	71.61 (1000.51)	6.01 (3.03)	64.42 (38.41)
Palmoplantar keratoderma	52	56.23 (42.34–74.67)	56.19 (2588.52)	5.69 (4.31)	51.68 (38.91)
Hepatic rupture	17	65.43 (39.71–107.82)	65.42 (977.09)	5.89 (3.09)	59.37 (36.03)
Food refusal	42	52.41 (38.25–71.80)	52.38 (1954.67)	5.60 (4.07)	48.44 (35.36)
Renal cell carcinoma	314	41.82 (37.30–46.89)	41.67 (11691.9)	5.29 (4.96)	39.15 (34.92)
Plantar erythema	19	58.79 (36.74–94.08)	58.78 (987.17)	5.75 (3.21)	53.86 (33.65)
Tumour rupture	32	51.15 (35.67–73.33)	51.13 (1454.82)	5.57 (3.78)	47.37 (33.04)
Hepatic encephalopathy	455	37.01 (33.66–40.69)	36.81 (14979.3)	5.12 (4.88)	34.84 (31.69)
Coma hepatic	43	39.46 (29.00–53.71)	39.44 (1516.40)	5.22 (3.90)	37.18 (27.32)
Hyperkeratosis	235	32.86 (28.81–37.47)	32.77 (6880.96)	4.96 (4.60)	31.20 (27.36)
Hepatic hydrothorax	6	66.44 (28.64–154.08)	66.43 (349.85)	5.91 (1.52)	60.20 (25.96)
Metastatic renal cell carcinoma	91	34.22 (27.71–42.26)	34.18 (2780.89)	5.02 (4.29)	32.48 (26.30)
Metastases to adrenals	39	34.01 (24.64–46.94)	34.00 (1185.14)	5.01 (3.71)	32.31 (23.41)
Alpha 1 foetoprotein decreased	3	70.12 (21.27–231.16)	70.12 (183.97)	5.98 (0.40)	63.21 (19.18)
Oesophageal varices haemorrhage	72	25.10 (19.83–31.77)	25.08 (1600.83)	4.59 (3.85)	24.16 (19.09)
Metastases to lung	321	22.10 (19.77–24.71)	22.02 (6224.85)	4.41 (4.16)	21.31 (19.06)
Metastases to diaphragm	4	52.59 (18.96–145.86)	52.59 (186.87)	5.60 (0.86)	48.62 (17.53)
Tumour necrosis	36	25.51 (18.28–35.60)	25.50 (814.47)	4.62 (3.42)	24.55 (17.59)

### Clinical subgroup analysis

**FIGURE 5:**
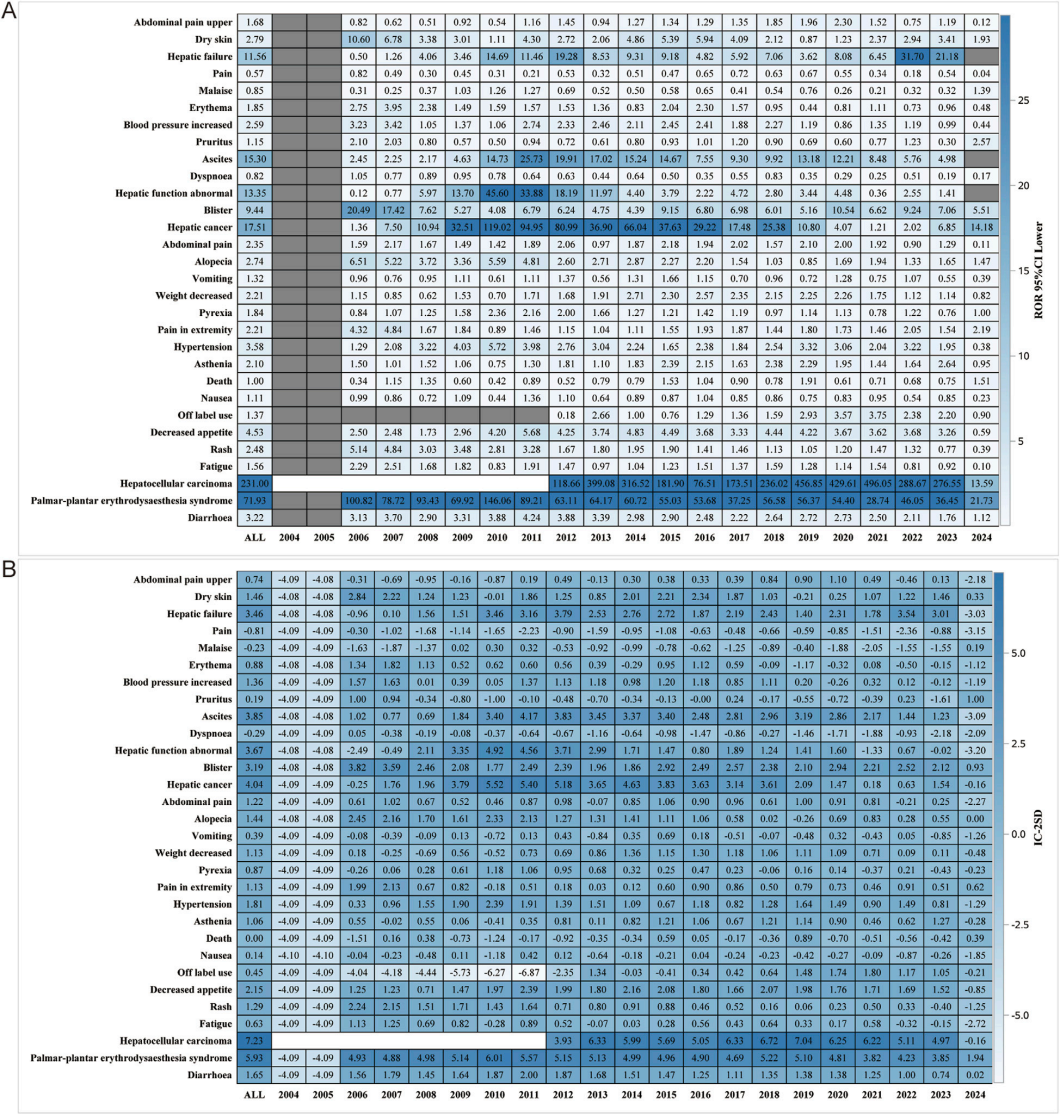
Report year subgroup analysis. **(A)** ROR; **(B)** BCPNN.

**FIGURE 6:**
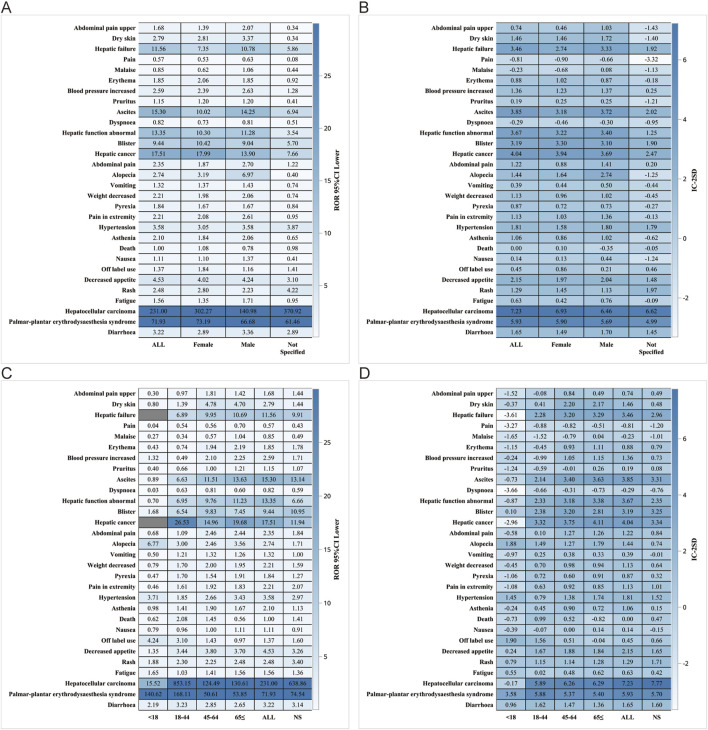
Gender subgroup analysis. **(A)** ROR; **(B)** BCPNN. Age subgroups. **(C)** ROR; **(D)** BCPNN.

**FIGURE 7:**
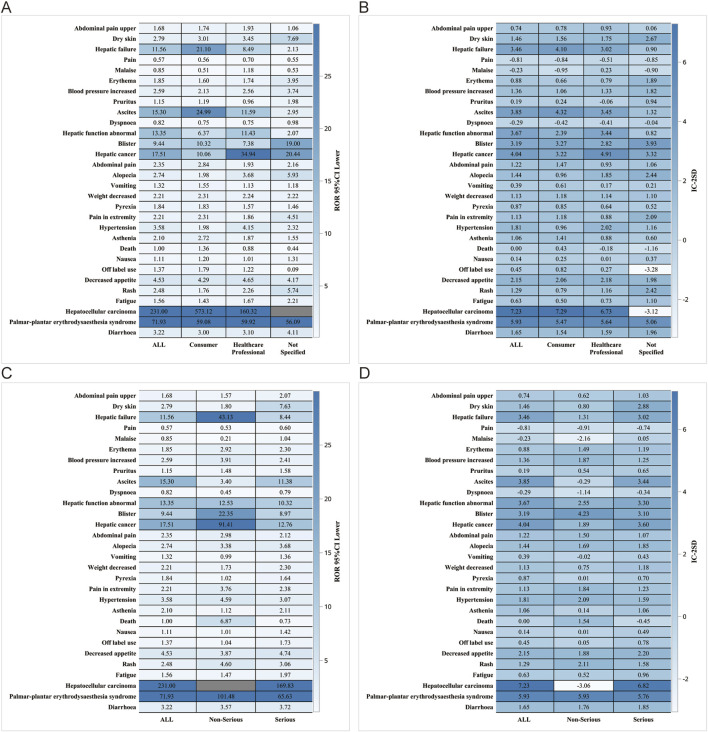
Reporter and Serious reporting subgroup analysis. Reporter subgroup **(A)** ROR; **(B)** BCPNN. Serious reporting subgroup. **(C)** ROR; **(D)** BCPNN.

We performed further detailed analyses by year of report, gender, age, reporter, and serious report subgroups, respectively; in terms of report year, the damage in the liver was still very severe, including HCC, hepatic cancer, hepatic function abnormal and hepatic failure (Figure 5). In terms of gender, the ROR (95% CI) results suggested that HCC was significantly higher in females than in males, with no significant difference in the rest of the cases (Figure 6A), whereas the IC (IC-2SD) results equitably showed no significant difference in AEs between genders (Figure 6B). The results of the age subgroups analysis showed that people aged 18–44 years were more likely to have an AE of HCC (Figure 6C). Palmar-plantar erythrodysaesthesia syndrome, on the other hand, was present in both subgroups of less than 18 and 18–44 years [ROR (95% CI) analysis results], whereas the IC (IC-2SD) results remained undetectable between the age subgroups (Figure 6D). In terms of reporters (Figure 7A), the ROR (95% CI) detected that consumers reported the highest number of AEs presented as HCC, as observes with hepatic failure, while hepatic cancer was more frequently reported by healthcare professionals, while the results for IC (IC-2SD) were not significantly different (Figure 7B). The ROR for serious reporting subgroups (95% CI) suggests greater number of AEs in the serious group for HCC, whereas the non-serious group had palmar-plantar erythrodysaesthesia syndrome, hepatic cancer, hepatic failure, and blisters (Figures 7C, D).

## Discussion

This study represents the first extensive and systematic pharmacovigilance investigation using the FAERS database to analyze Sorafenib-related post-marketing AEs. The primary objective of this study was to meticulously and comprehensively characterize, describe, and analyze the Sorafenib-related AEs reported to date. The findings presented here provide valuable and accurate insights into the safety of Sorafenib in real-world clinical settings.

This study found that Sorafenib-associated AEs occurred in a significantly higher proportion of males (66.89%) than females (26.30%), which also correlates with epidemiologic results (Forner et al., 2018). In terms of age, majority of patients were older than 65 years old (41.76%), which also reflects the fact that the current population with the disease is mainly older than 65 years (Hsieh et al., 2017; Boucai et al., 2024; Cappuyns et al., 2024). Physicians and consumers were the main reporters, accounting for 62.43% of all the total number of reports. This is mainly because the FAERS database constitutes a system of spontaneous reporting, with the most important part of the reporting being performed by physicians and consumers (Li et al., 2021). The number of reports was higher between 2009-2018, after which there was a significant downward trend. Moreover, as the use of the drug increases, the corresponding number of reports of AEs also increases. As the use of the drug has been perfected and the use of Sorafenib has been gradually mastered, the reports of AEs have begun to decrease.

The percentage of serious reports on Sorafenib use was as high as 88.50%. As a multikinase inhibitor, its mechanism of action dictates that it may affect multiple biological processes, leading to a variety of serious AEs (van Malenstein et al., 2013). The reason why the outcome is mainly “Hospitalization - Initial or Prolonged” may be due to the occurrence of some more serious adverse reactions that have to be hospitalized to be resolved. The early occurrence of AEs within the first 30 days after dosing is a key observation, suggesting the need for vigilant monitoring during this period. Sorafenib was administered concomitantly with Furosemide and Amlodipine, possibly because Sorafenib is an oral tyrosine kinase inhibitor (TKI) and inhibition of VEGF signaling interrupts angiogenesis and is associated with the development of hypertension and compensatory hypertrophy. Compensated hypertrophy eventually leads to heart failure requiring the involvement of Furosemide and Amlodipine (Ashton, 1965; Sanford and Keam, 2009; Hall et al., 2013).

In our study, the most common and important SOC-level AEs were gastrointestinal disorders, general disorders, and administration site conditions, and skin and subcutaneous tissue disorders, which were consistent with safety data from labeling and clinical trials. Among the many AEs, gastrointestinal disorders (represented by diarrhoea and decreased appetite) and skin and subcutaneous tissue disorders (represented by palmar-plantar erythema syndrome and rasherythrodysaesthesia syndrome) are the most frequent and significant. Several clinical trials have confirmed that Sorafenib is strongly associated with a higher incidence of diarrhoea, decreased appetite, and hand-foot skin reaction (HFSR) (Abou-Alfa et al., 2006; Escudier et al., 2007; Chang et al., 2022; Luo et al., 2022; Zheng et al., 2022). Chloracne, a rare but serious AE that may be caused by Sorafenib, has still not been explained in detail and rationality (Pickert et al., 2011; Cohen, 2015). HCC due to Sorafenib should be focused on probably because of the fact that only approximately 30% of patients can benefit from Sorafenib and that this population usually acquires resistance to the drug within 6 months (Chen and Xia, 2019). Recent studies have shown that epigenetics, transporter processes, regulatory cell death, and the tumor microenvironment play roles in the onset and progression of Sorafenib resistance in HCC(Shi et al., 2011; Zhang et al., 2013; Lachaier et al., 2014; Takahashi et al., 2014; Lou et al., 2015; Zhou et al., 2016; Chen and Xia, 2019; Li et al., 2019).

Our results suggest that poorly differentiated thyroid carcinoma is strongly associated with Sorafenib AEs, and we speculate that this may be related to the use of Sorafenib, a new class of TKIs, which causes tumors to become resistant to these anti-angiogenic treatments, partly due to compensatory mechanisms (Laursen et al., 2016). On the other hand, in patients with advanced HCC treated with Sorafenib, early Alpha 1 foetoprotein (AFP) increase can serve as a predictive biomarker of disease progression and poor overall and progression-free survival (OS and PFS) (Nakazawa et al., 2013). Sorafenib is the first targeted multikinase inhibitor and first-line chemotherapeutic agent approved for the treatment of RCC. Approximately 22% of patients with RCC do not respond to early Sorafenib treatment owing to intrinsic resistance, and the majority of the remaining patients develop Sorafenib resistance and tumor progression after 6–15 months of treatment, which has become a major obstacle to the efficacy of Sorafenib drug therapy (Bao et al., 2018; He et al., 2021). The main mechanisms of Sorafenib resistance in RCC are classified into five different categories: non-coding RNA-mediated resistance (Gao et al., 2014; Li et al., 2019), upregulation of pro-angiogenic signaling pathways (Schor-Bardach et al., 2009; Crona et al., 2019), activation of RAF/MEK/ERK and PI3K/AKT/mTOR pathways (Wilhelm et al., 2004; Sekino et al., 2020), abnormal intracellular pharmacokinetics (Edginton et al., 2016; Zhang et al., 2020), and tumor hypoxic microenvironment-mediated drug resistance (Tang et al., 2010; Micucci et al., 2015). Therefore, it is crucial to conduct numerous clinical and preclinical trials to help overcome Sorafenib resistance in patients with RCC. Currently, combination therapy with agents such as Sorafenib and sequential VEGFR-TKIs is the main approach for overcome Sorafenib resistance.

Hepatic encephalopathy, a complication of portal hypertension secondary to cirrhosis, can affect Sorafenib treatment (Chen et al., 2022). Previous studies have suggested a possible relationship between TKIs and portal hemodynamics in animal models or exploratory clinical trials with very limited samples (Allaire et al., 2021). Several patients can develop other common SOC levels of PT levels of AE, such as hypertension, hemorrhage, alopecia, suggesting that Sorafenib has a multi-systemic toxicity on metabolism and nutrition disorders, cardiac disorders, and blood and lymphatic system disorders. Currently, hypertension, HFSR, and fatigue are attributed to the inhibition of several tyrosine kinases, whereas haemorrhage, proteinuria, wound complications, and perforation are more closely related to the inhibition of the VEGF pathway (Roodhart et al., 2008; Hartmann et al., 2009; Keating and Santoro, 2009).

In our analysis, although most AEs were consistent with safety data obtained from labels and clinical trials, we identified other significant AEs that were not explicitly reported in regulatory trials, such as gait inability. It is worth noting that Sorafenib, as a multi-target kinase inhibitor, may affect multiple body systems and potentially cause a range of adverse reactions. Gait inability may be a secondary effect of certain AEs; for example, if patients experience severe fatigue or neurological issues, it may affect their walking ability. As for palmoplantar keratoderma and hyperkeratosis of the skin, this is a novel observation: after receiving TKI therapy, such as Sorafenib, two patients developed squamous cell carcinomas (SCC) and inflammation of AKs. Three patients developed keratoacanthomas. The development of actinic keratoses (AK) or SCC as a result of Sorafenib therapy was first reported by Lacouture et al. (2006), Kong et al. (2007). As with single kinase inhibitors, cutaneous AEs have been reported with Sorafenib, specifically a HFSR complicated by hyperkeratosis (Lountzis and Maroon, 2008) and is closely related to the spiny follicular hyperkeratosis eruption discovered by researchers Franck et al. (2010). Although these are not explicitly mentioned in the Sorafenib clinical guidelines, considering HFSR, there are similarities between palmoplantar erythrodysesthesia syndrome (PPES) and palmoplantar keratoderma (PPK) as well as hyperkeratosis in the affected body areas. However, the specific underlying mechanisms require further investigation. These findings suggest that for patients undergoing Sorafenib treatment, it is necessary to closely monitor indicators such as the nervous and mental systems, and skin changes, and to intervene symptomatically in a timely manner when needed. These findings emphasize the importance of continuous monitoring of drug-related adverse reactions and provide a valuable reference for informed decision making in drug selection. Several studies have focused on enhancing the therapeutic efficacy of Sorafenib in cancer treatment using innovative combinations and delivery strategies. Chen et al. demonstrated that combining 5-MTP with Sorafenib improves its efficacy against lung cancer. This combination not only reduces cell proliferation, but also hinders metastasis by downregulating key proteins, such as vimentin and MMP9, while affecting signaling pathways, such as Akt and STAT3, to enhance tumor inhibition (Chen et al., 2024). Xu et al. explored a self-activated cascade-responsive system that co-delivered Sorafenib with USP22 short hairpin RNA (shRNA). By silencing USP22, which plays a role in drug resistance, this co-delivery system sensitized HCC cells to Sorafenib, allowing more effective cancer cell apoptosis and improved overall treatment outcomes (Xu et al., 2021). Wang et al. discussed various advanced delivery methods for Sorafenib, such as nanoparticles and self-assembly systems, which were designed to improve stability, reduce side effects, and target tumor cells more precisely. These approaches are particularly promising for HCC and other cancers, highlighting the potential applications of Sorafenib beyond traditional chemotherapy (Wang et al., 2023).

This study capitalized on the inherent strengths of a large-scale real-world survey and employed sophisticated data mining techniques. However, it is crucial to acknowledge and address certain limitations that require careful consideration: 1) The FAERS database, which is a spontaneous reporting system, may lead to incomplete and inaccurate information collected from various countries and healthcare professionals, thereby introducing biases in the analysis. For instance, reporting bias and indication bias can make it challenging to determine whether AEs are drug-induced or a result of the progression of the underlying disease’s progression; 2) Due to their low incidence, very rare AEs associated with Sorafenib use may not be statistically significant in disproportionality calculations. Other safety signals may have not been identified yet; 3) The specificity of attributing AEs to Sorafenib is limited and may be influenced by concomitant medications; 4) The impact of Sorafenib dosage changes, renal impairment, hepatic dysfunction, and other internal/external factors over time cannot be fully explained; 5) Due to the lack of a total count that includes the entire population treated with Sorafenib, it is not possible to accurately calculate the incidence rate of each AEs; 6) Disproportionality analysis, which is used to identify statistical significance based on signal strength, cannot completely eliminate the confounding effects of drug-drug interactions.

## Conclusion

In summary, this study used real-world data from the FAERS database to conduct a comprehensive investigation and identify AEs associated with Sorafenib through disproportionality analysis. The AEs detected in this study were largely consistent with those listed in the product labes, and several potential AEs were also identified, including gait inability, palmoplantar keratoderma, and hyperkeratosis. Furthermore, this study reported the median onset time for labeled and off-label AEs, along with detailed subgroup analysis results, providing clinicians and pharmacists with a vigilant reference to optimize medication use and manage the safety issues associated with Sorafenib. Given the exploratory nature of our work, it is imperative to validate our findings in prospective studies and elucidate the underlying mechanisms and risk factors of AEs to explore their impact on drug utilization.

## Data Availability

The original contributions presented in the study are included in the article/Supplementary Material, further inquiries can be directed to the corresponding authors.

## Supplementary material

SUPPLEMENTARY FIGURE S1 Signal strength of reports of Sorafenib at the system organ class (SOC) level in the FAERS database. **(A)** SOC case; **(B)** SOC proportion.
