# Supplementary material for: A real-world pharmacovigilance study of Sorafenib based on the FDA Adverse Event Reporting System
Source: Front Pharmacol. 2024 Dec 17;15:1442765. doi: 10.3389/fphar.2024.1442765 (PMC11685139; doi:10.3389/fphar.2024.1442765)
Supplement: Supplementary file 3 [file Table2.docx]

**Supplement Table2**. Characteristics of cases of Sorafenib usage.

| Characteristics | Number of events (%) |
| --- | --- |
| Gender |  |
| Female(%) | 4899(26.30) |
| Male(%) | 12457(66.89) |
| Not Specified(%) | 1268( 6.81) |
| Age |  |
| <18(%) | 201( 1.08) |
| 18-44(%) | 892( 4.79) |
| 45-64(%) | 6147(33.01) |
| 65≤(%) | 7777(41.76) |
| NotSpecified(%) | 3607(19.37) |
| Report year |  |
| 2006(%) | 990( 5.32) |
| 2007(%) | 819( 4.40) |
| 2008(%) | 821( 4.41) |
| 2009(%) | 1158( 6.22) |
| 2010(%) | 1487( 7.98) |
| 2011(%) | 1642( 8.82) |
| 2012(%) | 1364( 7.32) |
| 2013(%) | 1006( 5.40) |
| 2014(%) | 1155( 6.20) |
| 2015(%) | 1300( 6.98) |
| 2016(%) | 1003( 5.39) |
| 2017(%) | 1312( 7.04) |
| 2018(%) | 1407( 7.55) |
| 2019(%) | 945( 5.07) |
| 2020(%) | 707( 3.80) |
| 2021(%) | 562( 3.02) |
| 2022(%) | 423( 2.27) |
| 2023(%) | 440( 2.36) |
| 2024(%) | 83( 0.45) |
| Reporter |  |
| Consumer(%) | 3977(21.35) |
| Not Specified(%) | 1520( 8.16) |
| Other health-professional(%) | 3307(17.76) |
| Pharmacist(%) | 2170(11.65) |
| Physician(%) | 7650(41.08) |
| Reported countries（ TOP ten) |  |
| United States of America(%) | 7453(40.02) |
| Japan(%) | 3842(20.63) |
| China(%) | 805( 4.32) |
| Brazil(%) | 580( 3.11) |
| Germany(%) | 567( 3.04) |
| France(%) | 565( 3.03) |
| Italy(%) | 510( 2.74) |
| Mexico(%) | 337( 1.81) |
| United Kiongdom(%) | 322( 1.73) |
| Korea(%) | 314( 1.69) |
| Indications（TOP ten) |  |
| Hepatocellular carcinoma(%) | 5140(27.60) |
| Hepatic cancer(%) | 4435(23.81) |
| Renal cell carcinoma(%) | 2225(11.95) |
| Not Specified(%) | 1318( 7.08) |
| Metastatic renal cell carcinoma(%) | 601( 3.23) |
| Product used for unknown indication(%) | 582( 3.13) |
| Thyroid cancer(%) | 517( 2.78) |
| Acute myeloid leukaemia(%) | 379( 2.04) |
| Renal cancer(%) | 331( 1.78) |
| Renal cell carcinoma stage IV(%) | 227( 1.22) |
| Serious report |  |
| Serious(%) | 16483(88.50) |
| Non-Serious(%) | 2141(11.50) |
| Outcome |  |
| Life-Threatening(%) | 880( 4.73) |
| Hospitalization - Initial or Prolonged(%) | 7146(38.37) |
| Disability(%) | 410( 2.20) |
| Death(%) | 4456(23.93) |
| Congenital Anomaly(%) | 3( 0.02) |
| Required Intervention to Prevent Permanent Impairment/Damage(%) | 13( 0.07) |
| Other(%) | 9564(51.35) |
| AE occurrence time—medication date (days) |  |
| 0-30d(%) | 5677(30.48) |
| 31-60d(%) | 1197( 6.43) |
| 61-90d(%) | 597( 3.21) |
| 91-120d(%) | 388( 2.08) |
| 121-150d(%) | 228( 1.22) |
| 151-180d(%) | 204( 1.10) |
| 181-360d(%) | 596( 3.20) |
| 360d<(%) | 578( 3.10) |
| Not Specified(%) | 9159(49.18) |
