# Supplementary material for: A real-world pharmacovigilance study of Sorafenib based on the FDA Adverse Event Reporting System
Source: Front Pharmacol. 2024 Dec 17;15:1442765. doi: 10.3389/fphar.2024.1442765 (PMC11685139; doi:10.3389/fphar.2024.1442765)
Supplement: Supplementary file 4 [file Table5.docx]

**Supplement Table 5**. The top 30 PTs with the highest signal frequency for Sorafenib positivity in ROR and BCPNN.

| **Preferred Terms** | **Case** | **ROR(95% CI)** | **IC(IC-2SD)** |
| --- | --- | --- | --- |
| Diarrhoea | 2756 | 3.35(3.22-3.48) | 1.70(1.65) |
| Palmar-plantar erythrodysaesthesia syndrome | 2109 | 75.29(71.93-78.80) | 6.04(5.93) |
| Hepatocellular carcinoma | 1791 | 244.01(231.00-257.76) | 7.44(7.23) |
| Fatigue | 1680 | 1.64(1.56-1.72) | 0.70(0.63) |
| Rash | 1548 | 2.61(2.48-2.75) | 1.36(1.29) |
| Decreased appetite | 1519 | 4.77(4.53-5.02) | 2.22(2.15) |
| Off label use | 1469 | 1.45(1.37-1.52) | 0.52(0.45) |
| Nausea | 1241 | 1.17(1.11-1.24) | 0.23(0.14) |
| Death | 1219 | 1.06(1.00-1.12) | 0.08(0.00) |
| Asthenia | 1124 | 2.23(2.10-2.37) | 1.14(1.06) |
| Hypertension | 1069 | 3.80(3.58-4.04) | 1.91(1.81) |
| Pain in extremity | 956 | 2.35(2.21-2.51) | 1.22(1.13) |
| Pyrexia | 915 | 1.96(1.84-2.10) | 0.96(0.87) |
| Weight decreased | 884 | 2.36(2.21-2.52) | 1.23(1.13) |
| Vomiting | 878 | 1.41(1.32-1.51) | 0.49(0.39) |
| Alopecia | 782 | 2.95(2.74-3.16) | 1.55(1.44) |
| Abdominal pain | 781 | 2.52(2.35-2.71) | 1.32(1.22) |
| Hepatic cancer | 725 | 18.85(17.51-20.31) | 4.18(4.04) |
| Blister | 720 | 10.16(9.44-10.94) | 3.31(3.19) |
| Hepatic function abnormal | 675 | 14.41(13.35-15.56) | 3.81(3.67) |
| Ascites | 646 | 16.55(15.30-17.90) | 4.00(3.85) |
| Pruritus | 615 | 1.24(1.15-1.34) | 0.31(0.19) |
| Blood pressure increased | 583 | 2.81(2.59-3.05) | 1.48(1.36) |
| Erythema | 572 | 2.01(1.85-2.18) | 1.00(0.88) |
| Hepatic failure | 514 | 12.62(11.56-13.78) | 3.62(3.46) |
| Dry skin | 503 | 3.05(2.79-3.33) | 1.60(1.46) |
| Abdominal pain upper | 500 | 1.84(1.68-2.01) | 0.87(0.74) |
| Skin exfoliation | 484 | 4.55(4.16-4.98) | 2.17(2.03) |
| Constipation | 477 | 1.72(1.57-1.88) | 0.77(0.64) |
| Hepatic encephalopathy | 455 | 37.01(33.66-40.69) | 5.12(4.88) |
