# Supplementary material for: A real-world pharmacovigilance study of Sorafenib based on the FDA Adverse Event Reporting System
Source: Front Pharmacol. 2024 Dec 17;15:1442765. doi: 10.3389/fphar.2024.1442765 (PMC11685139; doi:10.3389/fphar.2024.1442765)
Supplement: Supplementary file 5 [file Table3.docx]

**Supplement Table 3**. The top 30 PTs with the highest signal intensity for Sorafenib positivity in BCPNN.

| **Preferred Terms** | **Case** | **IC(95% CI)** |
| --- | --- | --- |
| Hepatocellular carcinoma | 1791 | 7.44(7.23-7.38) |
| Palmar-plantar erythrodysaesthesia syndrome | 2109 | 6.04(5.93-6.06) |
| Alpha 1 foetoprotein increased | 141 | 7.15(5.89-6.42) |
| Renal cell carcinoma | 314 | 5.29(4.96-5.29) |
| Hepatic encephalopathy | 455 | 5.12(4.88-5.16) |
| Hyperkeratosis | 235 | 4.96(4.60-4.98) |
| Liver carcinoma ruptured | 40 | 7.07(4.48-5.48) |
| Palmoplantar keratoderma | 52 | 5.69(4.31-5.13) |
| Metastatic renal cell carcinoma | 91 | 5.02(4.29-4.90) |
| Metastases to lung | 321 | 4.41(4.16-4.49) |
| Food refusal | 42 | 5.60(4.07-4.98) |
| Hepatic cancer | 725 | 4.18(4.04-4.26) |
| Ammonia increased | 149 | 4.37(3.95-4.43) |
| Coma hepatic | 43 | 5.22(3.90-4.80) |
| Amylase increased | 148 | 4.29(3.88-4.36) |
| Ascites | 646 | 4.00(3.85-4.09) |
| Oesophageal varices haemorrhage | 72 | 4.59(3.85-4.54) |
| Tumour rupture | 32 | 5.57(3.78-4.82) |
| Metastases to adrenals | 39 | 5.01(3.71-4.65) |
| Hepatic function abnormal | 675 | 3.81(3.67-3.89) |
| Tumour thrombosis | 23 | 6.39(3.61-4.86) |
| Thyroid cancer metastatic | 24 | 5.98(3.57-4.78) |
| Hepatic failure | 514 | 3.62(3.46-3.72) |
| Tumour necrosis | 36 | 4.62(3.42-4.39) |
| Lipase increased | 142 | 3.76(3.39-3.88) |
| Renal cell carcinoma stage IV | 18 | 6.63(3.29-4.72) |
| Protein induced by vitamin K absence or antagonist II increased | 17 | 7.51(3.26-4.81) |
| Plantar erythema | 19 | 5.75(3.21-4.56) |
| Blister | 720 | 3.31(3.19-3.40) |
| Hepatorenal syndrome | 41 | 4.07(3.16-4.06) |
