# Supplementary material for: A real-world pharmacovigilance study of Sorafenib based on the FDA Adverse Event Reporting System
Source: Front Pharmacol. 2024 Dec 17;15:1442765. doi: 10.3389/fphar.2024.1442765 (PMC11685139; doi:10.3389/fphar.2024.1442765)
Supplement: Supplementary file 7 [file Table6.docx]

**Supplement Table 6**. The top 30 PTs with the highest signal intensity for Sorafenib positivity in ROR and BCPNN.

| **Preferred Terms** | **Case** | **ROR(95% CI)** | **IC(IC-2SD)** |
| --- | --- | --- | --- |
| Hepatocellular carcinoma | 1791 | 244.01(231.00-257.76) | 7.44(7.23) |
| Poorly differentiated thyroid carcinoma | 4 | 631.12(157.84-2523.61) | 8.30(0.69) |
| Alpha 1 foetoprotein increased | 141 | 183.78(152.34-221.72) | 7.15(5.89) |
| Protein induced by vitamin K absence or antagonist II increased | 17 | 255.49(145.44-448.84) | 7.51(3.26) |
| Chloracne | 3 | 631.11(127.38-3127.02) | 8.30(0.18) |
| Liver carcinoma ruptured | 40 | 170.65(120.33-242.00) | 7.07(4.48) |
| Palmar-plantar erythrodysaesthesia syndrome | 2109 | 75.29(71.93-78.80) | 6.04(5.93) |
| Renal cell carcinoma stage IV | 18 | 117.13(70.83-193.72) | 6.63(3.29) |
| Reactive perforating collagenosis | 6 | 164.64(67.04-404.36) | 7.03(1.53) |
| Tumour thrombosis | 23 | 96.79(62.40-150.14) | 6.39(3.61) |
| Transcatheter arterial chemoembolisation | 5 | 131.49(50.17-344.62) | 6.77(1.23) |
| Thyroid cancer metastatic | 24 | 70.14(46.00-106.94) | 5.98(3.57) |
| Tumour embolism | 16 | 71.63(42.71-120.13) | 6.01(3.03) |
| Palmoplantar keratoderma | 52 | 56.23(42.34-74.67) | 5.69(4.31) |
| Hepatic rupture | 17 | 65.43(39.71-107.82) | 5.89(3.09) |
| Food refusal | 42 | 52.41(38.25-71.80) | 5.60(4.07) |
| Renal cell carcinoma | 314 | 41.82(37.30-46.89) | 5.29(4.96) |
| Plantar erythema | 19 | 58.79(36.74-94.08) | 5.75(3.21) |
| Tumour rupture | 32 | 51.15(35.67-73.33) | 5.57(3.78) |
| Hepatic encephalopathy | 455 | 37.01(33.66-40.69) | 5.12(4.88) |
| Coma hepatic | 43 | 39.46(29.00-53.71) | 5.22(3.90) |
| Hyperkeratosis | 235 | 32.86(28.81-37.47) | 4.96(4.60) |
| Hepatic hydrothorax | 6 | 66.44(28.64-154.08) | 5.91(1.52) |
| Metastatic renal cell carcinoma | 91 | 34.22(27.71-42.26) | 5.02(4.29) |
| Metastases to adrenals | 39 | 34.01(24.64-46.94) | 5.01(3.71) |
| Alpha 1 foetoprotein decreased | 3 | 70.12(21.27-231.16) | 5.98(0.40) |
| Oesophageal varices haemorrhage | 72 | 25.10(19.83-31.77) | 4.59(3.85) |
| Metastases to lung | 321 | 22.10(19.77-24.71) | 4.41(4.16) |
| Metastases to diaphragm | 4 | 52.59(18.96-145.86) | 5.60(0.86) |
| Tumour necrosis | 36 | 25.51(18.28-35.60) | 4.62(3.42) |
